# Inferring the Contribution of Microbial Taxa and Organic Matter Molecular Formulas to Ecological Assembly

**DOI:** 10.3389/fmicb.2022.803420

**Published:** 2022-02-18

**Authors:** Robert E. Danczak, Aditi Sengupta, Sarah J. Fansler, Rosalie K. Chu, Vanessa A. Garayburu-Caruso, Lupita Renteria, Jason Toyoda, Jacqueline Wells, James C. Stegen

**Affiliations:** ^1^Ecosystem Sciences, Pacific Northwest National Laboratory, Richland, WA, United States; ^2^Department of Biology, California Lutheran University, Thousand Oaks, CA, United States; ^3^Environmental Molecular Sciences Laboratory, Pacific Northwest National Laboratory, Richland, WA, United States

**Keywords:** community assembly, β-nearest taxon index, null modeling, FTICR-MS, metacommunity ecology, meta-metabolome ecology

## Abstract

Understanding the mechanisms underlying the assembly of communities has long been the goal of many ecological studies. While several studies have evaluated community wide ecological assembly, fewer have focused on investigating the impacts of individual members within a community or assemblage on ecological assembly. Here, we adapted a previous null model β-nearest taxon index (βNTI) to measure the contribution of individual features within an ecological community to overall assembly. This new metric, called feature-level βNTI (βNTI_feat_), enables researchers to determine whether ecological features (e.g., individual microbial taxa) contribute to divergence, convergence, or have insignificant impacts across spatiotemporally resolved metacommunities or meta-assemblages. Using βNTI_feat_, we revealed that unclassified microbial lineages often contributed to community divergence while diverse groups (e.g., Crenarchaeota, Alphaproteobacteria, and Gammaproteobacteria) contributed to convergence. We also demonstrate that βNTI_feat_ can be extended to other ecological assemblages such as organic molecules comprising organic matter (OM) pools. OM had more inconsistent trends compared to the microbial community though CHO-containing molecular formulas often contributed to convergence, while nitrogen and phosphorus-containing formulas contributed to both convergence and divergence. A network analysis was used to relate βNTI_feat_ values from the putatively active microbial community and the OM assemblage and examine potentially common contributions to ecological assembly across different communities/assemblages. This analysis revealed that P-containing formulas often contributed to convergence/divergence separately from other ecological features and N-containing formulas often contributed to assembly in coordination with microorganisms. Additionally, members of Family *Geobacteraceae* were often observed to contribute to convergence/divergence in conjunction with both N- and P-containing formulas, suggesting a coordinated ecological role for family members and the nitrogen/phosphorus cycle. Overall, we show that βNTI_feat_ offers opportunities to investigate the community or assemblage members, which shape the phylogenetic or functional landscape, and demonstrate the potential to evaluate potential points of coordination across various community types.

## Introduction

Evaluating the processes which govern community diversity is often the goal of ecological studies across all ecosystems (Swenson et al., 2006; Kraft et al., 2007; Gilbert and Bennett, 2010; Smith and Lundholm, 2010; Chase and Myers, 2011; George et al., 2011; Stegen et al., 2013; Herren and McMahon, 2017; Zhou and Ning, 2017; Danczak et al., 2020b). Analogously, researchers have also focused on understanding the processes governing the composition of organic molecules or metabolites within organic matter (OM) assemblages (Danczak et al., 2020a, 2021). While methods might vary in how researchers investigate these processes (e.g., variation partitioning, trait-based analyses, and null modeling), each study attempts to determine when, where, and how various ecological assembly processes give rise to specific community/assemblage configurations. By better understanding the distribution of these processes and the circumstances under which they dominate, we will be able to better understand the fundamental principles governing community/assemblage structure. However, less attention has been paid to the impact of ecological processes on individual community/assemblage members or to the impact of individual members on ecological assembly. Hereafter, in order to limit confusion, both biological and chemical members are referred to as “features,” while biological communities and OM assemblages are referred to as “communities” (Table 1).

**Table 1:** Table of terms used throughout the manuscript and their definitions.

Terms	Definitions
Feature	*Feature* is used liberally in this manuscript. This term is meant to describe anything that can be described in a relational dendrogram (e.g., microbial community members and FTICR-MS molecular formula).
Community	*Community* refers to a collection of ecological features. Here, that describes both microbial communities and organic matter assemblages.
Contribution	|βNTI_feat_| > 1, with significant contributions inferred when |βNTI_feat_| > 2; When a feature “contributes” to ecological assembly, it exerts some deterministic impact on the community.
Contribution to convergence	βNTI_feat_ < −2; Features that “contribute to convergence” are those which drive ecological/functional similarities across communities.
Contribution to divergence	βNTI_feat_ > 2; Features that “contribute to divergence” are those which drive ecological/functional differences across communities.

Evaluating the impacts of individual features provides several benefits to ecological researchers. Firstly, feature-level metrics provide researchers the opportunity to investigate how specific features (e.g., a microbial taxon or metabolite) contribute to assembly under varied environmental regimes or across different spatiotemporal scales. As detailed by Ning et al. (2020), members of a given taxonomic level can respond differently to environmental stresses, with some experiencing variable selection and others being limited by dispersal. Feature-level metrics will allow researchers to disentangle the contributions (or lack thereof) of individual members and identify groups putatively most relevant to community assembly. The ability to observe individual contributions would allow researchers to evaluate the ecological roles of specific organisms and/or organic molecules in the absence of explicit physiological or biochemical information based on traits inferred from phylogeny/taxonomy. For example, Ning et al. (2020) observed that a group of Bacillales significantly experienced homogeneous selection in hot/dry environments potentially due to their enhanced survivability.

Secondly, feature-level metrics will allow ecologists to compare and relate the contributions to assembly dynamics across community types in order to observe potential ecological coordination. For example, these metrics can be directly compared across different community types to find groups of cross-community features, which exert coordinated control on the larger metacommunity/meta-assemblage, potentially highlighting a common ecological pressure or ecological interaction (e.g., a specific environmental condition driving the selection of features across community boundaries). Investigating how assembly compares across disparate groups has enabled a deeper understanding of the fundamental factors structuring ecological communities. Danczak et al. (2020b) revealed that viral and microbial communities experienced coordinated assembly processes despite facing separate environmental pressures in a fractured shale ecosystem. Jiao et al. (2020) demonstrated that the balance of cross-kingdom species interactions across Archaea-Bacteria-Fungi mediated community assembly in an agricultural soil ecosystem. These examples indicate that the ability to measure and relate feature-level contributions to ecological assembly will help identify components disproportionally impacting phylogenetic or functional community structure.

We propose that a new metric called feature-level β-nearest taxon index (βNTI_feat_) based upon an existing null modeling framework (βNTI) will provide these benefits. βNTI is particularly capable in assessing the assembly dynamics associated with ecological metacommunities and OM meta-metabolomes/assemblages and we show that it can be adapted to feature-level analyses. βNTI has been used extensively to study assembly processes. For example, researchers have revealed relationships between microbial community development and organic matter degradation (Stegen et al., 2016, 2018), coordination of assembly between viral and microbial communities (Danczak et al., 2020b), the balance of niche- and dispersal-based processes the soybean microbiome (Moroenyane et al., 2021), and emphasized the importance of salinity in the assembly of desert microbial communities (Zhang et al., 2019).

Recently, Ning et al. (2020) developed an iterative null model based on β-net relatedness index (βNRI) called iCAMP, which represents a potential route to identify these feature-level dynamics. By first identifying the minimum phylogenetic level at which a phylogenetic signal exists (e.g., a relationship between evolutionary history and niche occupancy; Blomberg et al., 2003; Stegen et al., 2012), iCAMP groups community members and measures the ecological pressures acting upon that level. Using these metrics calculated across the phylogenetic tree, iCAMP can then estimate the balance of assembly processes acting on the community. This method is an excellent way to account for phylogenetic groups experiencing varied assembly processes in whole community analyses and represents a novel way to follow sub-community assembly through time or space. However, while capable of identifying assembly processes at levels below the entire community, this approach still investigates processes impacting assembly at the subcommunity level rather than measuring the degree which an individual feature impacts or is impacted by assembly. Fodelianakis et al. (2021) instead took an approach, called “phyloscore analysis” that focuses instead on the ecological contributions of specific taxa within a microbial community. Likewise, βNTI_feat_ focuses on individual features to measure their ecological contribution to community dynamics and highlights a point of complementarity across community, subcommunity, and feature-level foci (Table 1).

Feature-level β-nearest taxon index provides insight into the degree to which each observed feature contributes to either ecological convergence or divergence (Table 1). Ecological convergence occurs when some feature drives similarities in the phylogenetic or functional landscape across samples or within a dataset. Such a feature would be more phylogenetically or functionally conserved than expected by random chance. In contrast, ecological divergence occurs when a feature drives phylogenetic or functional differences across samples or a dataset; these features are more divergent than expected by random chance. Based on these interpretations, βNTI_feat_ stands as the phylogenetic or dendrogram-based complement to taxonomic metrics like SIMPER (Clarke, 1993). Importantly, given that βNTI_feat_ does not rely on abundance or taxonomic-based distance metrics, it is able to overcome many of the limitations associated with SIMPER (Warton et al., 2012). When compared to the phyloscore metric described by Fodelianakis et al. (2021), it is much more similar though differs in some mathematical specifics (namely the null implementation) and in its application across different scales.

Here, we describe the theory behind the βNTI_feat_ calculations, apply it to microbial and environmental metabolomic data, and discuss how interpretations vary with scale and dataset. First, we reveal that βNTI_feat_ can identify microbial taxa (down to the amplicon sequence variant, or ASV, level) and environmental metabolites (down to the specific molecular formulas), which disproportionally contribute to the ecological structure of the respective community. Second, we demonstrate that we can track features with disproportionate contributions through time and relate them to each other to uncover ASVs and molecular formulas that have coordinated contributions to the biological and chemical composition of the study system.

## Materials and Methods

### Sample Collection

Detailed sample collection is outlined in Sengupta et al. (2021a) but will be described briefly here. Sediments were collected from the hyporheic zone of the Columbia River shoreline in eastern Washington state on 14 January 2019 at 9 a.m. Pacific Standard Time. Five samples were collected from within a meter range, combined to make a composite sediment sample, and then sieved on site through a 2 mm sieve into a glass beaker. Sieved sediment was kept on blue ice for 30 min until transported back to the lab, where it was stored at 4°C until experimentation.

### Experimental Design

A detailed experimental design is outlined in Sengupta et al. (2021a) but will be described briefly here. Sieved sediment was partitioned into two sets of vials, each vial containing 10 g of sediment: one set of vials were under inundated conditions for 23 days, and the other set were allowed to dry for 23 days. Once the vials were permitted to acclimate, they were subject treatment regimes designed around a series of wet/dry transition periods. While the details are important, these regimes largely translate to two bulk treatments based upon the number of days left dry: cumulatively dry (34, 31, and 27 days) and cumulatively inundated (0, 4, and 8 days). This resulted in a total of 20 vials per cumulative treatment.

### 16S rRNA Gene Sequencing and Processing

Detailed DNA and cDNA extraction methods can be found in Sengupta et al. (2021a). Briefly, incubated sediments were centrifuged and flash-frozen. gDNA was extracted following the protocol established by Bottos et al. (2018) RNA was extracted using the Qiagen PowerSoil RNA extraction kit (Qiagen, Germantown, MD), treated for contaminate DNA using DNase, quantified using a Qubit RNA kit (Thermo Fisher, Waltham, MA), and reverse transcribed into cDNA using the SuperScript™ IV First-Strand Synthesis System (Thermo Fisher Scientific, Waltham, MA). Amplicon sequencing for both gDNA and cDNA was performed following The Earth Microbiome Project. Sequences are accessible at NCBI’s Sequence Read Archive using the accession number # PRJNA641165.

Sequence processing was performed using QIIME2 (Bolyen et al., 2019). Raw amplicon sequences were imported into the QIIME2 environment, denoised using DADA2 (*q2-dada2*), and assigned taxonomy using the SILVA v138 database (*q2-feature-classifier*; Quast et al., 2013; Callahan et al., 2016; Bokulich et al., 2018). A phylogenetic tree was generated by first aligning amplicons using the MAFFT aligner (Katoh, 2002) and then generating a maximum-likelihood tree (*q2-phylogeny*). The 16S rRNA gene amplicon maximum-likelihood tree is stored as Supplementary File 1.

### FTICR-MS Analysis

Detailed Fourier transform ion cyclotron resonance mass spectrometer (FTICR-MS) data acquisition is outlined in Danczak et al. (2020a) and Sengupta et al. (2021a) but will be described briefly here. In brief, a solid-phase extraction on a PPL cartridge (Bond Elut) was used to concentrate carbon and remove salt (Dittmar et al., 2008). Extracted samples were injected into a 12 Tesla (12T) Bruker SolariX FTICR-MS outfitted with a standard electrospray ionization source (ESI) configured to negative mode. Resulting spectra were processed using the BrukerDaltonik Data Analysis software (v4.2) to obtain a peak list; Formularity was then used to assign molecular formulas to detected peaks following the Compound Identification Algorithm (Kujawinski and Behn, 2006; Tolić et al., 2017). The report generated in Formularity was then processed using the ftmsRanalysis R package to calculate various molecular properties (e.g., double-bond equivalents, modified aromaticity index, nominal oxidation state of carbon, Kendrick’s defect, etc.) and assign compound classes (Hughey et al., 2001; Kim et al., 2003; Koch and Dittmar, 2006; LaRowe and Van Cappellen, 2011; Bailey et al., 2017; Rivas-Ubach et al., 2018; Bramer et al., 2020). Using the methods outlined in Danczak et al. (2020a), we generated a molecular characteristics dendrogram (MCD) for use in dendrogram-informed analyses; the MCD is stored as Supplementary File 2.

### Ecological Analyses

Multivariate differences across the microbial community and OM assemblage were detected using ordinations in combination with PERMANOVA statistics (*adonis*; vegan package v2.5-7; Oksanen et al., 2019). A principal coordinate analysis (PCoA; *pcoa*; ape package v5.5) was generated using both Bray–Curtis dissimilarity (*vegdist*; vegan package v2.5-7) and β-mean nearest taxon distance (βMNTD; *comdistnt*; picante package v1.8.2) were calculated for both the microbial community and OM assemblage (Kembel et al., 2010; Oksanen et al., 2019; Paradis and Schliep, 2019).

### The βNTI_feat_ Calculation

**Figure 1:**
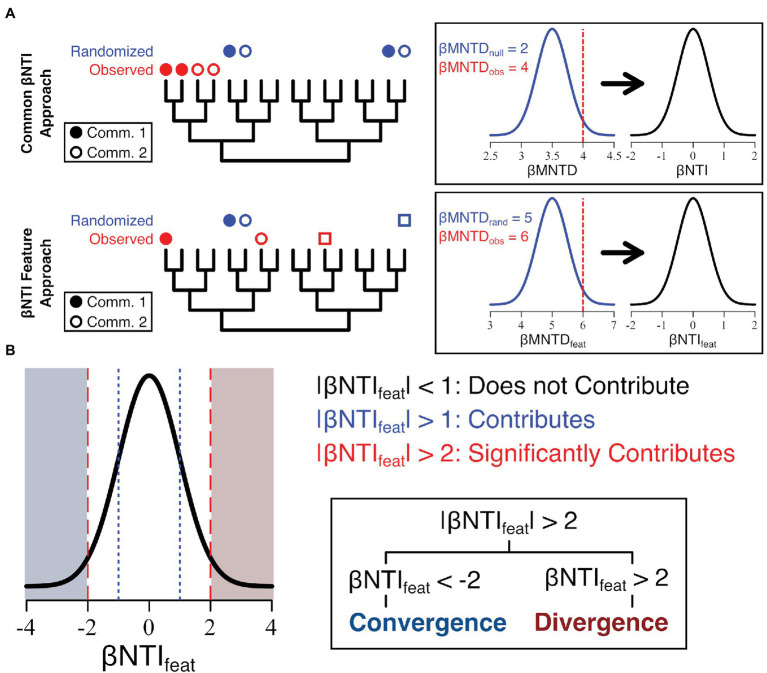
Conceptual depiction of feature-level β-nearest taxon index (βNTI_feat_) calculation as it compares to the common β-nearest taxon index (βNTI) calculation **(A)** and the subsequent interpretation **(B)**. Essentially, the βNTI_feat_ calculation mirrors βNTI though instead measures the distance between a given focal feature in one community/assemblage (closed circles) and the nearest member in another community/assemblage (open circles and squares).

The βNTI_feat_ is intrinsically linked to the βNTI calculation and helps us understand the relationship between observed dendrogram-based relationships of individual features and some null expectation (Figure 1). First, βMNTD_feat_, the minimum relational distance of a feature in one community to the nearest feature in another, needs to be calculated for the observed community across the entire dataset:


(1) $$\beta {\mathrm{MNTD}}_{feat}=\frac{1}{n}\overset{n}{\underset{j=1}{\sum }}f_{a_{i}}\min\left(d_{a_{i}b_{j}}\right)\;$$

where $f_{a_{i}}$ is the relative abundance of feature *a* in community *i*, *n* is the number of communities/samples in the dataset, and $\min\left(d_{a_{i}b_{j}}\right)$ is the average minimum relational distance (e.g., the distance between tips on a dendrogram—equivalent to phylogenetic distance) of the fixed feature *a* in the fixed community *i* to any feature *b* in all communities *j*. In these current analyses, we are allowing conspecifics in our calculations, but they can be excluded pending experimental design. In this case, a conspecific feature is one which is present across both halves of a pairwise comparison and the inclusion/exclusion implementation matches that of the *comdistnt* in the picante R package (Kembel et al., 2010). In practice, this metric measures the average minimum distance between a given feature in one community and all other features in other communities. The key departure from the standard βMNTD calculation is that this calculation occurs from a fixed perspective; only one community is compared to all other communities at a single time. As with the traditional βNTI calculation, βMNTD_feat_ was also calculated for 999 randomized communities, which were generated by shuffling the tips of the provided dendrogram/phylogenetic tree using the function *taxaShuffle* from the picante R package (Kembel et al., 2010). Additionally, the null βMNTD_feat_ calculation has small amounts of phylogenetic noise (e.g., 1 × 10^−20^–5 × 10^−20^) injected into them to allow for features present across both halves of the pairwise comparison to be included. By combining the null results with our observed results, we can calculate βNTI_feat_:


(2) $$\beta {\mathrm{NTI}}_{feat}=\frac{\beta {\mathrm{MNTD}}_{feat}^{obs}-\bar{\beta {\mathrm{MNTD}}_{feat}^{null}}}{\beta {\mathrm{MNTD}}_{feat}^{sd}}\;$$

where $\beta {\mathrm{MNTD}}_{feat}^{obs}$ is the observed βMNTD_feat_ measurement, $\bar{\beta {\mathrm{MNTD}}_{feat}^{null}}$ is the average βMNTD_feat_ for the null results, and $\beta {\mathrm{MNTD}}_{feat}^{sd}$ is the SD of $\beta {\mathrm{MNTD}}_{feat}^{null}$ values.

### Estimating Feature-Level Ecological Dynamics

Following the same philosophy underlining the typical βNTI interpretations, βNTI_feat_ seeks to quantify the ecological processes occurring within and across communities. When describing the underlying theory of βNTI_feat_, we use the term “community” as the general term to describe any assemblage of ecological data (Table 1). However, we want to stress that this method can be used on any relational set of data (e.g., microbial communities and DOM assemblages). Unlike βNTI, which evaluates whole communities through space or time, βNTI_feat_ is focused on identifying the contributions to community assembly by individual community members (Figure 1). For example, while βNTI is well-suited to identify variable selection as a process driving differences between two communities, it cannot measure which community members may be driving that variable selection. In contrast, βNTI_feat_ has been adapted from βNTI to track the influence of individual features, which significantly contribute either to convergence or divergence.

Feature-level β-nearest taxon index can be calculated in three separate ways in order to account for variations in scale (e.g., a complete experiment vs. sub-groupings within the experiment). First, it can be calculated for an entire dataset yielding a single βNTI_feat_ value for each feature within a dataset. This approach is particularly useful in identifying which features in the whole dataset contribute to convergence or divergence. Second, it can be calculated within groups, for example, if you have a factor with two levels, you can calculate βNTI_feat_ for each of those levels. Results from this type of analysis will generate a βNTI_feat_ value for each feature within each level of the factor (i.e., if there are two factors and 100 features, 200 βNTI_feat_ values will be created) and is well-suited to compare differential feature contribution. Finally, βNTI_feat_ can be calculated truly pairwise (akin to the standard βNTI calculation) in order to provide complete spatial or temporal resolution at the feature level. By performing the βNTI_feat_ analysis on a fixed focal sample (a 0 day dry sample here), you can get a true temporal perspective. Importantly, all these calculations can be done with absolute abundance, relative abundance, or presence/absence data.

The interpretation of the βNTI_feat_ is directly analogous to the interpretation of βNTI but focuses on individual “features” instead. Here, a “feature” is any member of a community or assemblage that is subject to ecological pressures (a microorganism or environmental metabolite, for example; Table 1). |βNTI_feat_| < 1 means that a feature has an insignificant contribution to ecological variation across the metacommunity or meta-assemblage, 1 < |βNTI_feat_| < 2 indicates that a feature somewhat contributes ecological variation, and |βNTI_feat_| > 2 suggests that a given feature significantly contributes to ecological variation. These patterns can be further resolved based upon the sign of βNTI_feat_. When βNTI_feat_ trends negative (e.g., <−1), we suggest that the feature contributes to convergence within the scale of analysis. Under this definition, these are features (or groups of related features) that significantly drive relational commonalities across a given analytical scale. For example, these features could represent a phylogenetically conserved niche of microorganisms or consistent group of molecular formula that are disproportionally impacted by selective processes. When βNTI_feat_ instead trends positive (e.g., >1), we suggest that the feature contributes to divergence. Under this definition, these are features (or groups of related features) that drive relational differences across an analytical scale. These could be those microorganisms which arose to prominence under varied environmental conditions (e.g., they were selected for/against) or those organic matter constituents that were produced/consumed under specific conditions.

### Network Analysis

Weighted gene co-expression network analysis (WGCNA) was used to relate βNTI_feat_ results and identify modules of related contributions to ecological assembly within community types (e.g., within the putative active community or molecular formula assemblage) or across community types (e.g., the putatively active community compared to the molecular formula assemblage; WGCNA package v1.70-3; Langfelder and Horvath, 2008). Networks were first generated in R and then visualized/analyzed using Cytoscape v3.8.2 (Shannon et al., 2003). Specifically, βNTI_feat_ values for the putatively active ASVs (as identified *via* cDNA amplicon data) were related to the βNTI_feat_ values for the molecular formulas. As part of this analysis, we identify modules of interconnected features (either ASVs or formula). These modules represent those features which have either positive or negative relationships in their contributions to ecological or functional convergence/divergence. Additional ecological metrics were calculated for modules containing both ASVs and molecular formulas including Shannon’s diversity (H; *diversity*, vegan package v2.5-7), exp(H) per recommendation from Jost (2006), Pielou’s evenness (J), number of taxonomic Orders within a module, and the number of ASVs in a module (e.g., richness) The relative abundance of elemental composition groups was also calculated for these modules. The Cytoscape network file is stored as Supplementary File 3.

### Data and Code Availability

Fourier transform ion cyclotron resonance mass spectrometer data are available on the ESS-DIVE archive at https://data.ess-dive.lbl.gov/view/doi:10.15485/1807580, sequence data are available on the NCBI Sequence Read Archive PRJNA641165, and scripts used throughout this manuscript (including various versions of the βNTI_feat_ calculation) can be found on GitHub at https://github.com/danczakre/betaNTI-feature (Sengupta et al., 2021b).

## Results and Discussion

### βNTI_feat_ Revealed That Uncultured Microbial Lineages Differentially Contribute to Ecological Processes Across Total and Putatively Active Communities

**Figure 2:**
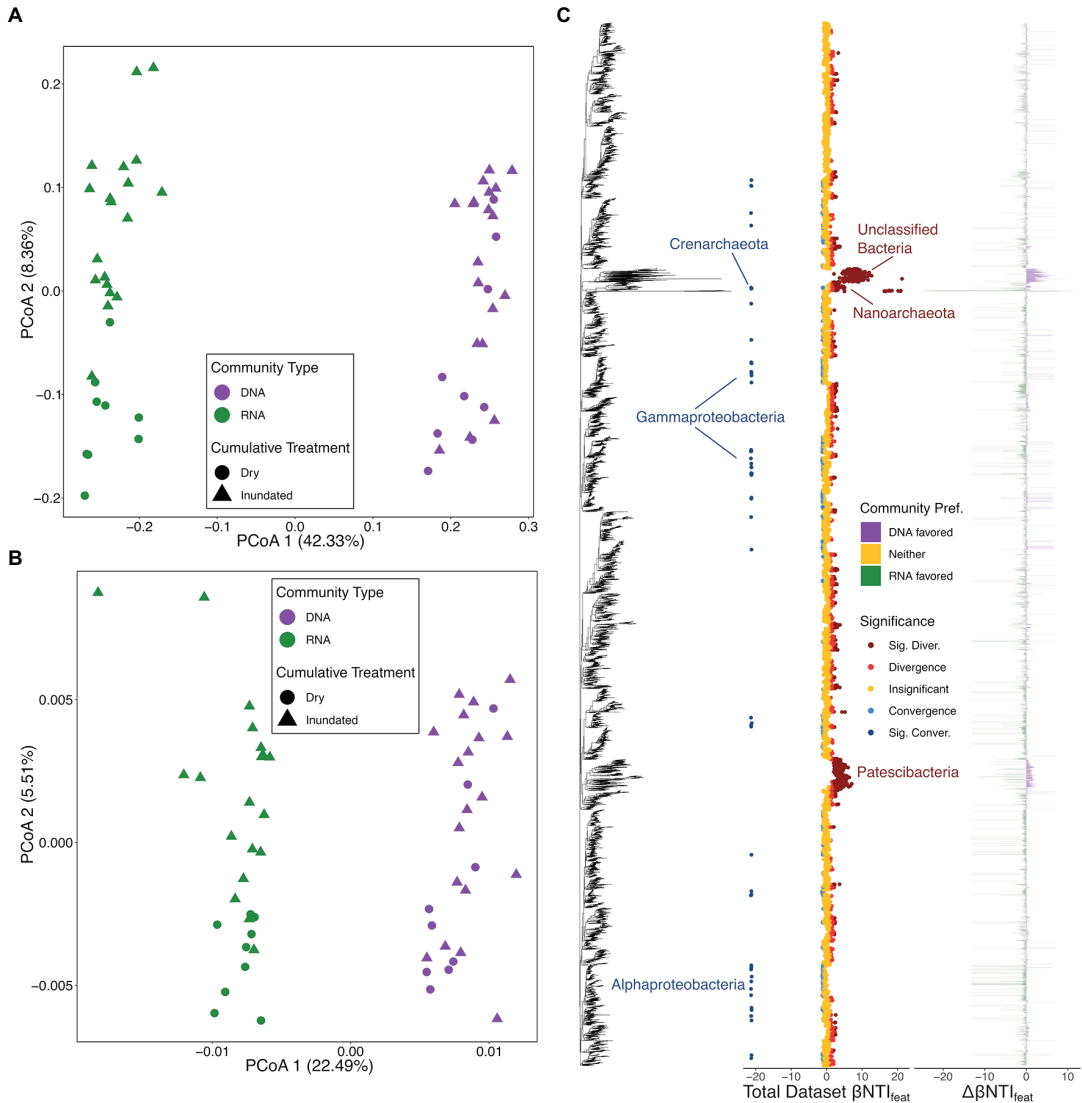
Microbial ordinations and overview of microbial feature-level β-nearest taxon index (βNTI_feat_) results. **(A)** Bray–Curtis dissimilarity-based principal coordinate analysis (PCoA) with colors representing community type, the total community defined by 16S rRNA gene amplicon results (DNA) and transcribed 16S rRNA gene amplicon results (RNA), and shapes distinguishing treatment type. **(B)** β-mean nearest taxon distance (βMNTD)-based PCoA with a legend matching panel **(B)**. **(C)** βNTI_feat_ results consisting of three panels: the 16S rRNA gene amplicon tree, βNTI_feat_ values for the whole dataset with colors indicating contribution to ecological assembly (Direction), and the difference in βNTI_feat_ values across the total microbial community and putatively active community (Community Pref.). Under the Direction legend, “Insignificant” represent |βNTI_feat_| < 1, “Convergence” or “Divergence” indicate 1 < | βNTI_feat_| < 2, and “Sig. Conver.” or “Sig. Diver.” represent |βNTI_feat_| > 2. Under the Community Pref. legend, “DNA favored” indicates that the given microbial group had a higher absolute βNTI_feat_ value in the total community, “RNA favored” indicates that the given microbial group had a higher absolute βNTI_feat_ value in the putative active community, and “Neither” indicates no difference between communities. Microbial groups discussed throughout the manuscript are called out where applicable.

We first analyzed the overall microbial dynamics and observed that the total community (using 16S rRNA gene sequencing) and putatively active community (*via* transcribed 16S rRNA sequencing) were significantly divergent from each other (Figures 2A,B). A Jaccard-based PCoA of Family-level taxonomic assignments revealed that not only were the two community types divergent, but also the communities under cumulatively dry treatments were divergent from those under inundated treatments (DNA – PERMANOVA Pseudo-F: 1.4826, value of *p* < .05; RNA – Pseudo-F: 3.4025, value of *p* < .001; and Type – PERMANOVA Pseudo-F: 38.653, value of *p* < .001; Figure 2A). Similar cross treatment dynamics were also apparent once sample similarity was analyzed using phylogenetic relatedness (e.g., βMNTD; DNA – PERMANOVA Pseudo-F: 1.8191, value of *p* < .05; RNA – Pseudo-F: 3.7559, value of *p* < .01; and Type – PERMANOVA Pseudo-F: 69.54, value of *p* < .001; Figure 2B). Given that the largest differences existed between the total microbial community and the putatively active component (based upon Pseudo-F statistic for the Type comparison), this information was used as a baseline of differences to interpret downstream analyses.

When compared to existing taxonomic metrics (e.g., SIMPER), βNTI_feat_ allows researchers to identity microbial groups impacting ecological structure rather than composition providing insight into community development (Clarke, 1993). In turn, we can evaluate the degree to which specific microbes contribute to the breadth of ecological strategies contained within metacommunities. This goes far beyond information on variation in taxonomic composition or standard diversity metrics. Existing approaches can identity cross-community differences in the abundance of a given taxon, but cannot identify the assembly processes leading to those differences in abundance. For each taxon (or molecule), βNTI_feat_ quantifies the assembly processes it experiences, the degree to which it influences ecological structure, and elucidates the environmental conditions that lead to variation in these influences and contributions.

Feature-level β-nearest taxon index revealed that varied microbial lineages differentially impacted assembly of the total microbial community and the putatively active microbial community (Figure 2C). Looking at the βNTI_feat_ dynamics across both the total and putative active communities, we see that members of an unclassified/uncultured group of Bacteria, members of the Patescibacteria (intermingled with some Class Bacilli), and members of the Nanoarchaeota consistently contribute to ecological divergence (Figure 2C). Those taxa which contribute to ecological convergence; however, are less conserved and more widespread across the phylogenetic tree with ASVs appearing within the Crenarchaeota, Alphaproteobacteria, and Gammaproteobacteria (Figure 2C).

Given that the largest community differences existed between the total and putatively active communities (Figures 2A,B), we focused on evaluating which microbial groups differentially contributed across these groups. We see that those members of the uncultured bacterial group and the Patescibacteria are among the most notably differential. Specifically, while many members appear to contribute to either convergence or divergence across both communities, we see that fewer members of these lineages contribute to assembly within the putatively active community. Out of 364 detected Patescibacteria across all microbial data, 305 contributed to assembly (|βNTI_feat_| > 1) in the total community, while only 34 impacted the putatively active community (Figure 2C). This pattern demonstrates that the uncultured microbial lineages contribute more to the phylogenetic structure of the total community than the active community. The stronger role played by uncultured lineages in the total community may indicate that these taxa have a background role and are not as relevant to the ecology of the active community.

**Figure 3:**
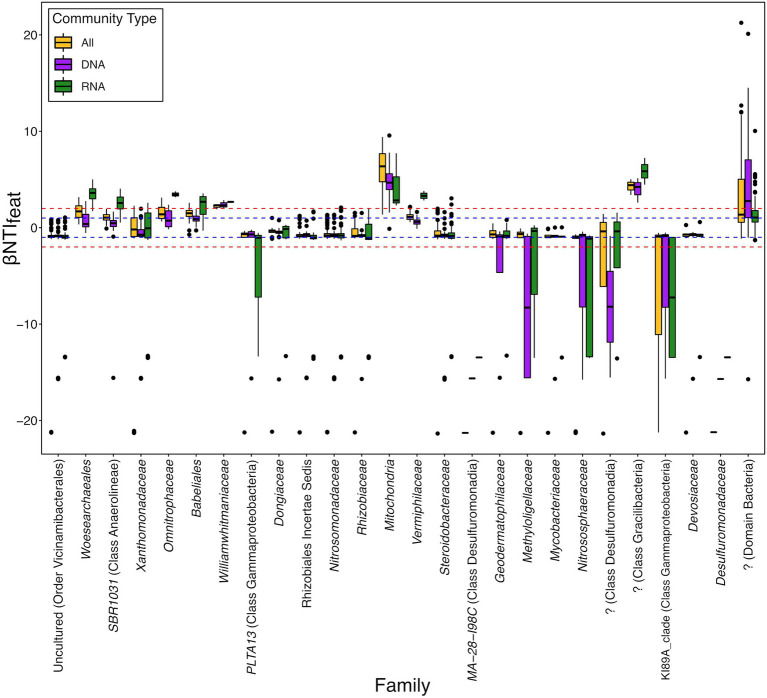
Distribution of feature-level β-nearest taxon index (βNTI_feat_) values for ASVs belonging to the Family-level taxonomic groups which contribute to either convergence or divergence on average. Some of these taxonomic groups only have a single ASV (e.g., Family MA-28-I98C), whereas other groups have many more (the top two were the unclassified Bacteria with 454 ASVs and the Nitrosomonadaceae with 147 ASVs). “DNA” represents the βNTI_feat_ values for the total community (defined by the 16S rRNA gene amplicon results), “RNA” represents the βNTI_feat_ values for the putatively active community (defined by the transcribed 16S rRNA gene amplicon results), and “All” represents βNTI_feat_ values calculated from the complete community (both DNA and RNA combined). The blue dashed line at +1 and −1 represents the “Contributes” threshold outlined in Figure 1, while the red dashed line at +2 and −2 represents the “Significantly Contributes” threshold.

Breaking the data down based upon Family-level taxonomic groups that, on average, contributed to either convergence or divergence supported these broad phylogenetic patterns and we observed that contributions could be related to inferred functional potentials and spanned a range of relative abundances (Figure 3; Supplementary Figure 1). For example, an unknown family from Class Desulfuromonadia significantly drives convergence (βNTI_feat_ < −2) in the total community (DNA) but not in either the overall dataset (All) or putatively active (RNA) communities (Figure 3). Conversely, the Family *Woesearchaeales* significantly drives ecological divergence (βNTI_feat_ > 2) within the putatively active community as compared to the total community or overall dataset. In the case of these example taxonomic groups, we hypothesize that this is likely tied to their respective inferred functional potentials. Class Desulfuromonadia tend to feature sulfate reducing bacteria, while described members of Family *Woesearchaeles* are fermentative (Castelle et al., 2015; Waite et al., 2020). Given that the experimental design allowed these sediments to maintain oxic conditions, this would act as a selective force against sulfate reducers due to thermodynamic constraints but not necessarily fermentative organisms (i.e., members could still form syntrophic relationships). This would translate to a diminished presence within the putative active community and thereby prevent them from significantly contributing to either convergence or divergence and suggests that these organisms might be detected in the DNA dataset as either dormant cells or relic DNA (Lennon et al., 2018).

### Homologous Series Disproportionately Contribute to Convergence Regardless of Environment Type

**Figure 4:**
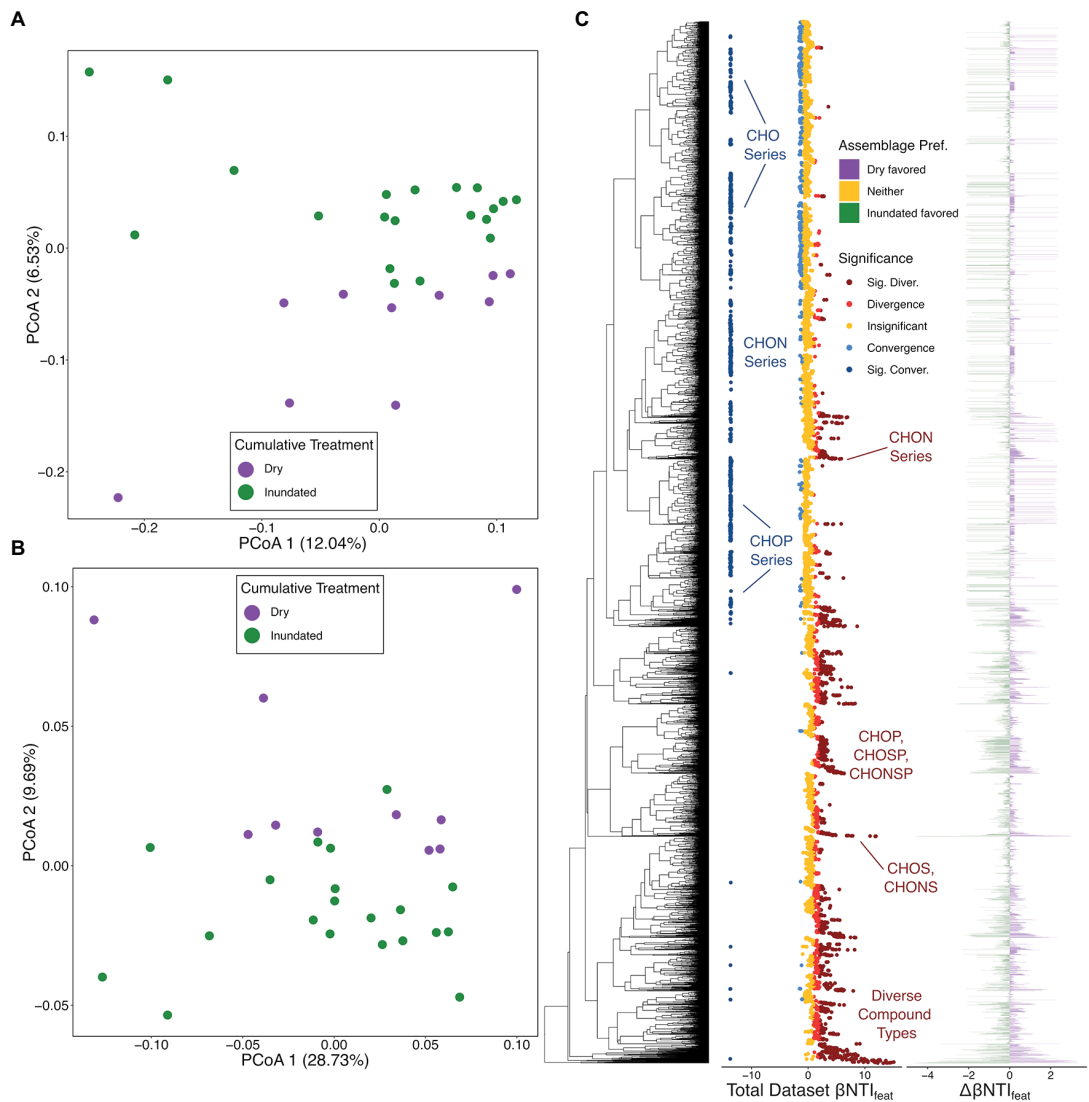
Organic matter (OM) ordinations and overview of OM feature-level β-nearest taxon index (βNTI_feat_) results. **(A)** Jaccard dissimilarity-based principal coordinate analysis (PCoA) with colors representing treatment type. **(B)** β-mean nearest taxon distance (βMNTD)-based PCoA with a legend matching panel **(B)**. **(C)** βNTI_feat_ results consisting of three panels: the molecular characteristics dendrogram (MCD), βNTI_feat_ values for the whole dataset with colors indicating contribution to ecological assembly (Direction), and the difference in βNTI_feat_ values across the dry-treatment OM assemblage and inundated-treatment OM assemblage (Assemblage Pref.). Under the Direction legend, “Insignificant” represent |βNTI_feat_| < 1, “Convergence” or “Divergence” indicate 1 < |βNTI_feat_| < 2, and “Sig. Conver.” or “Sig. Diver.” represent |βNTI_feat_| > 2. Under the Assemblage Pref. legend, “Dry favored” indicates that aa given molecular formula had a higher absolute βNTI_feat_ value within dry assemblages, “Inundated favored” indicates that a given molecular formula had a higher absolute βNTI_feat_ value in inundated assemblages, and “Neither” indicates no difference between assemblages. Organic matter groups discussed throughout the manuscript are called out where applicable.

The overall DOM patterns largely mirror those of the putatively active microbial community. Namely, both Jaccard- and βMNTD-based analyses revealed that significant differences existed between the organic matter from dry and inundated samples (PERMANOVA Pseudo-F: 1.5085, value of *p* < .05; Figures 4A,B). Using this information, we calculated βNTI_feat_ for the combined dataset, the dry-only dataset, and the inundated dataset.

Looking first at the complete dataset, we see a higher proportion of features significantly contributing to functional divergence or convergence within the organic matter dataset (45.08%) than in the ASV dataset (20.99%). This potentially points simultaneously to the more transitive nature of many environmental metabolites as compared to microbial community members (Schmidt et al., 2011; Graham et al., 2018; Coward et al., 2019), while also highlighting the consistent role that some conserved organic matter constituents play. Specifically, we see many larger molecular formula (e.g., C47H68O10, C34H63O4P, etc.) and some molecular formula with more complex compositions (e.g., C46H68NO4S2P, C42H52NO9S2P, etc.) significantly contribute to divergence across the entire dataset (Figure 4C). This might suggest that these more complex compounds may play variable roles depending on the situation; they could be a hallmark of differential nutrient limitation (i.e., the functional needs of the meta-assemblage are highly variable; Graham et al., 2018; Garayburu-Caruso et al., 2020; Danczak et al., 2021) or highlight divergent degradation potential (e.g., common metabolites might be processed through different pathways).

Whereas divergence was driven by larger or more complex molecular formula compositions, we see that CHO-based homologous series (e.g., C23H30O14, C24H30O14, etc.), N-containing homologous series (e.g., C18H15NO10, C19H17NO10), and some P-containing homologous series (e.g., C17H25O9P, C18H27O9P) often contribute to convergence across the entire dataset. CHO-only molecular formulas are the most frequently observed types of organic matter from sedimentary and aquatic sources (Garayburu-Caruso et al., 2020) suggesting that these types of compounds could drive functional similarities across organic matter assemblages. As with those compounds which contribute to divergence, the contribution to convergence by the N-containing and P-containing formulas may also point to conserved functional needs or pathways (i.e., N/P-limitation drives selection for N/P-containing formulas, or ongoing metabolisms continually cycling these formulas).

Following the patterns established *via* the Jaccard- and βMNTD-based PCoAs, we evaluated the differences in environmental metabolites which contribute to convergence and divergence across the dry and inundated samples (Figure 4C). Unlike the ASV data, where groups contributed differently across the total and putatively active communities, we see a balanced set of contributions from formulas derived from dry and inundated organic matter with no clear discernable patterns.

**Figure 5:**
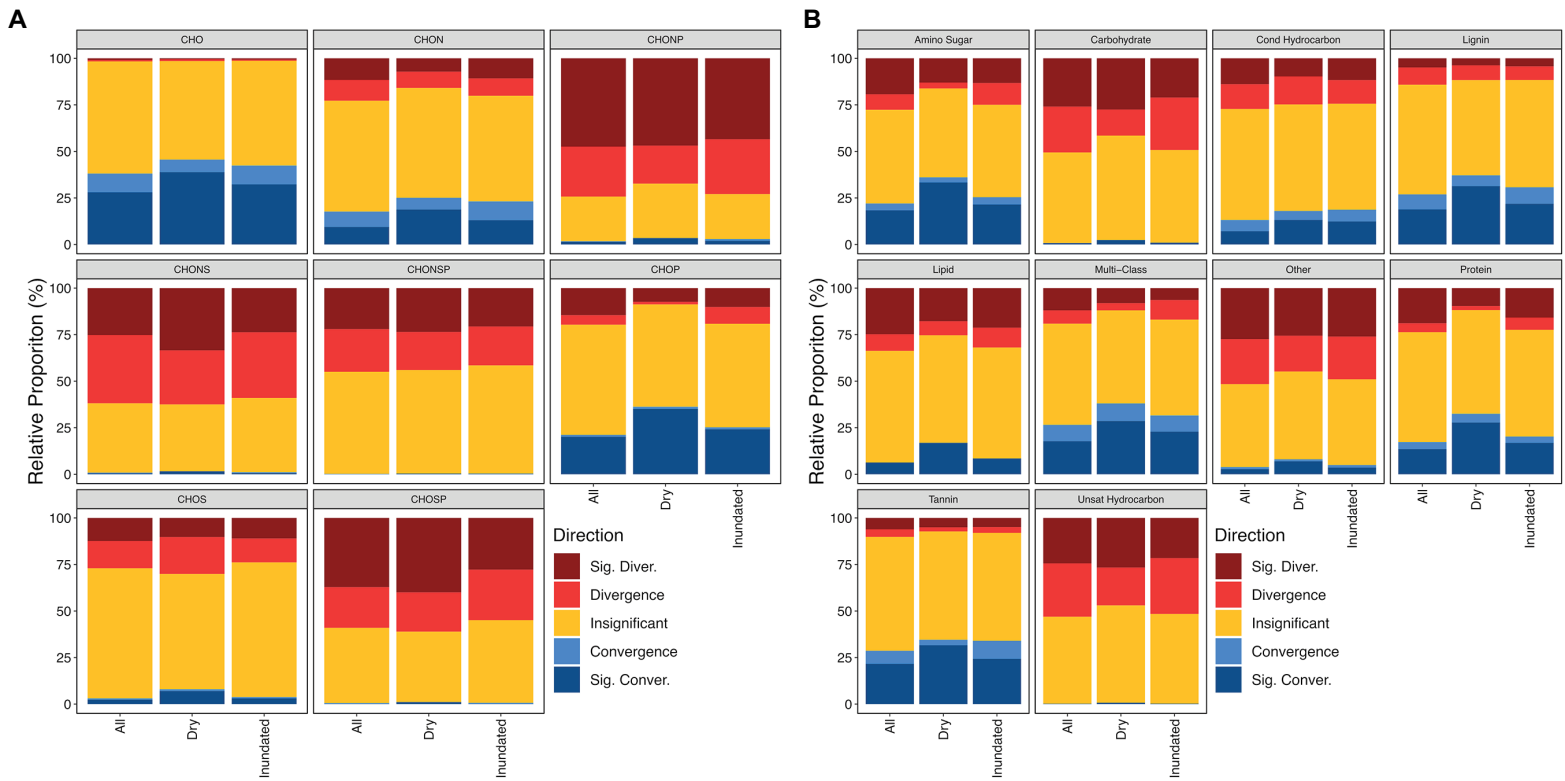
Proportion of contributions across different elemental composition groups **(A)** and compound classes **(B)**. These graphs outline the frequency that we observe a given contribution type within a given grouping. For example, out of all CHO molecular formulas within dry assemblages, we see that they contribute to convergence more frequently than CHONSP molecular formulas.

Grouping molecular formula by either elemental composition categories or compound classes further revealed little consistent variation across wet-dry conditions suggesting that the OM meta-assemblage might be more impacted by ecosystem than treatment condition in this case (Figure 5). Similar patterns were observed in a headwater stream, where bulk environmental properties were conserved across ecosystem types despite divergent OM assemblages undergoing variable selection as a result of hypothesized thermodynamic redundancy (Danczak et al., 2021). Briefly, thermodynamic redundancy is like functional redundancy, where compositionally divergent OM assemblages have similar thermodynamic properties. Here, βNTI_feat_ helps us examine whether this hypothesized redundancy exists across groups contributing to community structure. For example, in contrast to the thermodynamic behavior observed in Danczak et al. (2021), we see that the nominal oxidation state of carbon (NOSC) significantly varies across those CHON and CHOP containing molecular formula which contribute to convergence/divergence (Mann–Whitney U test value of *p* < .05; Supplementary Figure 2). Specifically, we observe that CHOP formulas with less inferred structural complexity (e.g., lower aromaticity index and double-bond equivalents) and more inferred lability (e.g., lower NOSC) drove significant convergence; differences in the properties of CHON formulas appear to manifest more as variation in distributions (e.g., those formulas which contribute to convergence are more constrained). Formulas featuring other elemental compositions exhibit less consistent behavior. This pattern suggests that thermodynamic restrictions potentially help dictate the contributions of the N/P-containing homologous series to the overall OM assemblage and may indicate that thermodynamic redundancy is not at play in this particular system. In other words, the preference for certain N/P-containing formulas might be dictated by organisms targeting the most preferential carbon source rather than some other limitation.

These results also demonstrate the capability for βNTI_feat_ to uncover functional dynamics within OM assemblages and assign ecological importance in the absence of abundance information.

### Network Analyses Reveal Groups of Molecular Formula and Microbes Which Contribute to Ecosystem Dynamics in a Coordinated Manner

While the previous sections focused on either analyzing complete datasets or subgroups within the overall dataset, βNTI_feat_ also provides sample-level information for each feature within a given dataset (Supplementary Figure 3). Using this approach, we can evaluate how the contribution of each feature to community assembly changes through either time or space by utilizing a single focal sample (here ECA_0Cyc_R2). Looking at the five most variable taxonomic groups from the putatively active community, we can see that different groups respond differently through treatment regimes (Supplementary Figure 3). For example, families DTB120, MVP-15, and order Myxococcocales rarely if ever cross the +2 βNTI_feat_ threshold for contributing to divergence. In contrast, the families *Williamwhitmaniaceae* and *Woesearchaeales* consistently cross that threshold. In contrast to these highly dynamic ASV taxa, the groups defined by a molecular formula’s elemental composition are less variable across samples (Supplementary Figure 3).

Having established that βNTI_feat_ can track dynamics through time, we believe that this metric is well-suited in network analyses. By performing a WGCNA using βNTI_feat_ values for each feature—as opposed to abundances—we can obtain modules of putatively active ASVs and OM formulas. These modules should reflect features which have either positive or negative relationships among their contributions to ecological/functional convergence and divergence. Within-cluster relationships between ASVs and organic molecules do not indicate co-variance in abundances (as in a traditional network analysis). Instead, these relationships indicate linkages between the ecology of microbes and the functional properties of organic molecules. We propose that these putative ecology-function linkages are more likely to indicate causal connections between microbes and molecules than networks built on relative abundances. There are nonetheless multiple potential interpretations of within-cluster relationships, leading to additional questions. For example, could the ASVs be responsible for degrading the linked molecular formulas? If a module contains exclusively molecular formulas, does this point a single pathway or do they represent an ecological interaction among pathways? By revealing features that have coordinated impacts on the ecological or functional structure of their respective community, network analyses can highlight important organisms or molecules. A better understanding of the relationships among ecological pressures impacting members across communities within a metacommunity will enable researchers to develop transitive principles regarding the coordination of ecological assembly (e.g., the mechanisms impacting cross trophic level assembly, etc.).

**Figure 6:**
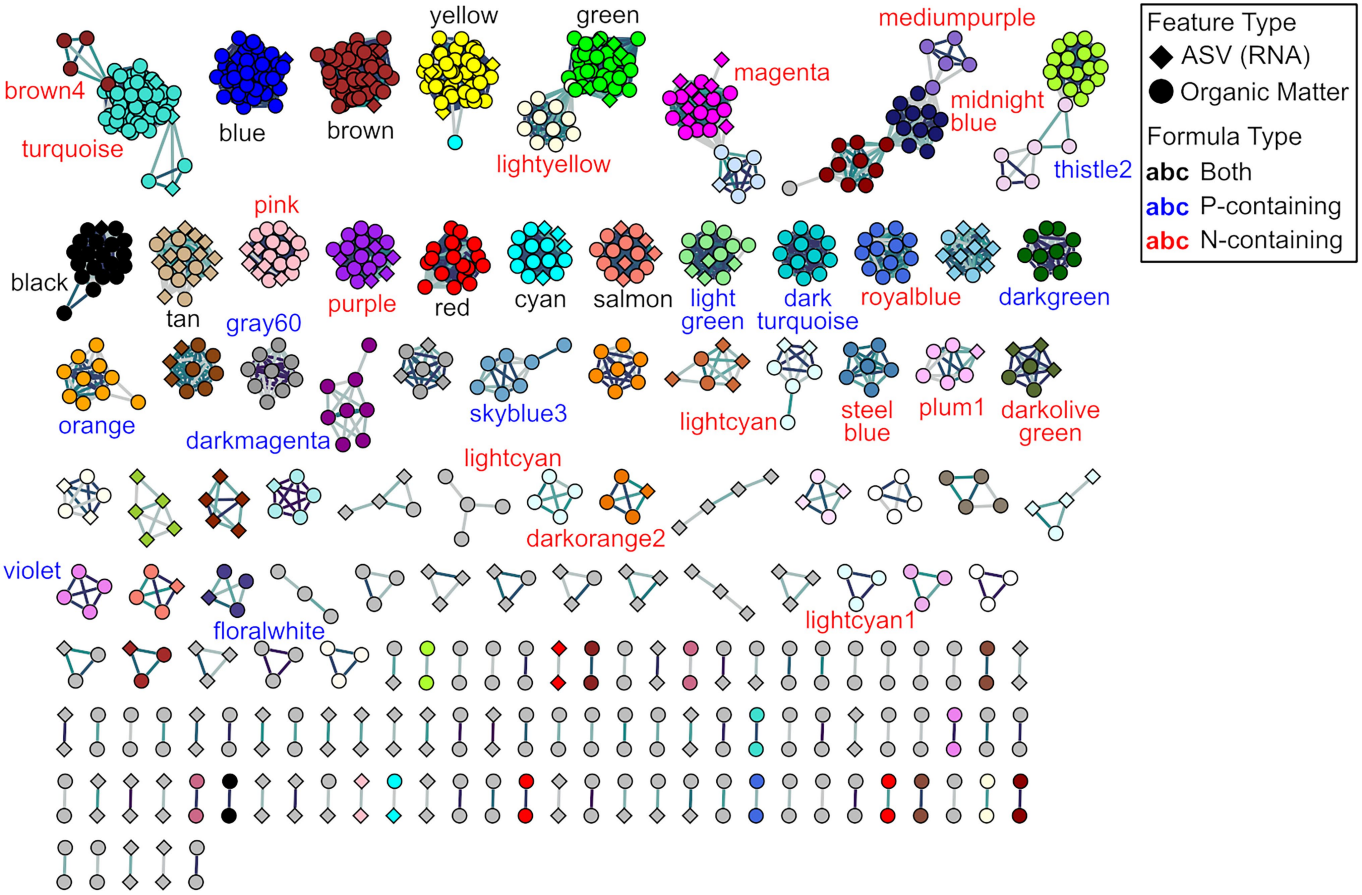
Feature-level β-nearest taxon index (βNTI_feat_)-based weighted gene co-expression network analysis (WGCNA). Modules with labels represent those modules which contain N- and/or P-containing molecular formulas. Node colors indicate the different modules identified as part of the WGCNA pipeline, node shapes represent different feature types, and label colors indicate whether the modules contain either N- and/or P-containing molecular formulas.

The network analysis resulted in the creation of 54 modules, with 28 modules having some mixture of ASVs and organic matter, two featuring exclusively ASVs, and 24 containing only molecular formulas (Figure 6; Supplementary Figure 4). Of these 54 modules, two modules were removed because the consisted only of doublets (e.g., modules where one feature related to only one other single feature). Focusing on the 27 modules which have a mixture of ASVs and molecular formulas, we observe a range of microbial diversity: for example, we observe that the gray and tan modules have the greatest number of different taxonomic groups (Supplementary Figure 4A). These modules also exhibit a range of characteristics across their molecular formulas: we see that the gray module predominantly contains CHO-only formulas, while the tan module primarily contains CHON formulas (Supplementary Figure 4B). This approach now allows us to evaluate which factors and taxonomic groups are coordinated in driving ecological dynamics.

Given the importance of nitrogen and phosphorus homologous uncovered in the metabolite βNTI_feat_ analyses, we examined modules containing at least two N- or P-containing formulas and contained more than two nodes. There were 16 modules with two or more N-containing molecular formulas, 10 modules with two or more P-containing formulas, and nine modules with both two or more N- and P-containing formulas (Figure 6). Analyzing the membership of these modules first revealed that the P-containing modules only featured four ASVs across all 10 modules, with three modules comprised exclusively of P-containing modules. This pattern suggests that these P-containing molecular formulas potentially experience a set of selective pressures distinct from other metabolites and ASVs. This characteristic could explain the disproportionate effect that phosphorus homologous series had on the functional structure of the OM meta-assemblage. In contrast, N-containing modules featured broader microbial and molecular diversity indicating that N-containing molecular formulas experience a more common set of pressures than P-containing formulas. As hypothesized above, this might be driven by nutritional requirements or some sort of pathway regulation. Finally, five different ASVs from Family *Geobacteraceae* were detected in three out of the nine modules featuring both N- and P-containing molecular formulas. While we do not have direct functional information due to the limits of amplicon sequencing, members of this family have reported roles in both the nitrogen cycle (*via* nitrate reduction, nitrification, and nitrogen fixation) and the phosphorus cycle (*via* aggressive phosphate acquisition; Naik et al., 1993; N’Guessan et al., 2010; Ueki and Lovley, 2010; Wang et al., 2018; Samaddar et al., 2019; Campeciño et al., 2020). Taken together, this potentially points to a combinatorial effect, whereby the functional development driven by N/P-containing formulas is tied to organismal selectivity (i.e., roles of *Geobacteraceae* in the N/P cycles).

## Conclusion

Feature-level β-nearest taxon index is a novel metric that enables researchers to investigate the contributions to ecological convergence and divergence with a given metacommunity or meta-assemblage. Using βNTI_feat_, we revealed that uncultured lineages contribute significantly to the ecological structure of microbial communities when assayed using 16S rDNA, but contributed little to the ecology of putatively actives communities assayed with 16S rRNA. These differences suggest that while these uncultured lineages represent a significant proportion of the microorganisms in some ecosystems (Brown et al., 2015; Anantharaman et al., 2016; Danczak et al., 2017; León-Zayas et al., 2017), they may be relatively minor contributors to the ecological composition of active microbes in the study system. This inference is complementary to traditional inferences based on relative abundances or taxonomic richness. That is, βNTI_feat_ quantifies the contribution of individual ASVs to the assembly of multi-dimensional ecological niche space occupied by communities, and how those contributions vary through space, time, and environmental conditions.

Applying βNTI_feat_ to OM provides analogous inferences, but instead quantifies the contribution of individual molecules to the assembly of the multi-dimensional functional space occupied by assemblages of organic molecules. In our study, we observed that OM homologous series primarily containing nitrogen or phosphorus differentially contributed to OM functional composition across the whole dataset. However, we do not see any noteworthy differences across wet-dry treatments indicating a stronger ecosystem-level control. Network analyses relating putatively active ASV βNTI_feat_ values to metabolite βNTI_feat_ values revealed formation of 54 distinct modules exhibiting coordinated contributions to their respective communities. Looking specifically at modules featuring N/P-containing molecular formulas, we observed that P-containing formulas appear to be largely disconnected from other metabolites and microorganisms, N-containing formulas are more integrated across feature types, and modules with both formula types exhibit a high incidence of *Geobacteraceae* members.

These dynamics potentially point to two larger scale hypotheses. First, the ecological contributions of P-containing formulas are often distinct from other metabolites indicating that they might occupy a unique functional space (at least compared to N-containing formulas). Second, certain microbial groups may offer contributions to convergence or divergence in concert with specific metabolite dynamics (here *Geobacteraceae* related to N/P-containing formulas). While these hypotheses still need to be independently verified, they point to specific conclusions that can be drawn from the use of βNTI_feat_. As further research uses this metric, more broad scale, transferrable hypotheses can be generated and help us better understand community assembly.

## Data Availability Statement

Publicly available datasets were analyzed in this study. This data can be found at: FTICR-MS data are available on the ESS-DIVE archive at: https://data.ess-dive.lbl.gov/view/doi:10.15485/1807580 and sequence data are available on the NCBI Sequence Read Archive PRJNA641165.

## Author Contributions

RD and JS conceptualized and designed the study. AS assisted in experimental design. JT, JW, LR, RC, SF, and VG-C performed the experiment. RD analyzed the data. RD and JS drafted the manuscript and all authors contributed to further writing. All authors contributed to the article and approved the submitted version.

## Funding

The initial experimental stages of this work were supported by the PREMIS Initiative at the Pacific Northwest National Laboratory (PNNL) with funding from the Laboratory Directed Research and Development Program at PNNL, a multi-program national laboratory operated by Battelle for the United States Department of Energy under Contract DE-AC05-76RL01830. The later stages of this work (e.g., data analysis, conceptual interpretation manuscript development) were supported by the United States Department of Energy-BER program, as part of an Early Career Award to JS at PNNL. A portion of the research was performed using EMSL, a DOE Office of Science User Facility sponsored by the Office of Biological and Environmental Research.

## Conflict of Interest

The authors declare that the research was conducted in the absence of any commercial or financial relationships that could be construed as a potential conflict of interest.

## Publisher’s Note

All claims expressed in this article are solely those of the authors and do not necessarily represent those of their affiliated organizations, or those of the publisher, the editors and the reviewers. Any product that may be evaluated in this article, or claim that may be made by its manufacturer, is not guaranteed or endorsed by the publisher.
