# Supplementary material for: Inferring the Contribution of Microbial Taxa and Organic Matter Molecular Formulas to Ecological Assembly
Source: Front Microbiol. 2022 Feb 18;13:803420. doi: 10.3389/fmicb.2022.803420 (PMC8894727; doi:10.3389/fmicb.2022.803420)
Supplement: Supplementary file 2 [file Data_Sheet_2.docx]

Supplementary Material for “Inferring the contribution of microbial taxa and organic matter molecular formulas to ecological assembly”

# Supplementary Data

**Supplemental File 1**: A maximum-likelihood 16S rRNA gene amplicon phylogenetic tree generated during amplicon processing in QIIME2.

**Supplemental File 2**: A molecular characteristics dendrogram (MCD) generated from the assigned molecular formulas identified using FTICR-MS.

**Supplemental File 3:** A Cytoscape file containing the weighted gene co-expression analysis (WGCNA) performed to investigate potential coordination in contributions to assembly across the putatively active microbial community and the organic matter assemblage.

# Supplementary Figures

## Supplementary Figures

**Supplementary Figure 1:** Plotting the relative abundances of ASVs belonging to the Family-level taxonomies listed on the x-axis. These taxonomies were selected to mirror those listed in Figure 3 of the main text which details the Family-level groups disproportionally contributing to convergence or divergence.

**Supplementary Figure 2:** Comparison of various molecular formula properties for those molecular formulas which either significantly contributed to convergence or divergence (|βNTI_feat_| > 2). DBE stands for double-bond equivalents, modified AI is the modified aromaticity index, and NOSC is the nominal oxidation state of carbon.

**Supplemental Figure 3:** βNTI_feat_ values plotted in a sample/time-resolved manner based upon a focal sample (ECA_0Cyc_R2 – 0 days dry); the number of cycles (e.g., 0Cyc, 1Cyc, etc.) denote increasing time scales outlined in the Materials and Methods. The blue dashed line at +1 and -1 represents the “Contributes” threshold outlined in **Figure 1**, while the red dashed line at +2 and -2 represents the “Significantly Contributes” threshold. a) Average βNTI_feat_ values for microbial Families with highly variable contributions depicted through by sample, through time. b) Average βNTI_feat_ values for all possible elemental compositions. The shaded backgrounds represent different sets of replicates while the x-label color denotes whether the replicates were cumulatively dry (red) or cumulatively inundated (black).

**Supplemental Figure 4:** Various types of metrics for modules containing both ASVs and molecular formulas. a) Diversity measurements for the microbial constituents in modules identified in the WGCNA network. Each diversity metric is denoted in the gray bar to the right, with the ‘Order Count’ representing the number of different microbial orders in a given module. Modules were arranged based upon the proportion of microbes present within the module. b) Relative abundance of a given elemental composition group in modules identified in the WGCNA network. Modules were arranged based upon the proportion of microbes present within the module.
